# Monkeypox: Is the world ready for another pandemic?

**DOI:** 10.3389/fpubh.2022.1001155

**Published:** 2022-10-13

**Authors:** Usman Abubakar Haruna, Shuaibu Saidu Musa, Emery Manirambona, Don Eliseo Lucero-Prisno, Antonio Sarría-Santamera

**Affiliations:** ^1^Department of Medicine, School of Medicine, Nazarbayev University, Astana, Kazakhstan; ^2^Global Health Focus Africa, Abuja, Nigeria; ^3^Department of Nursing Science, Ahmadu Bello University, Zaria, Nigeria; ^4^College of Medicine and Health Sciences, University of Rwanda, Kigali, Rwanda; ^5^Department of Global Health and Development, London School of Hygiene and Tropical Medicine, University of London, London, United Kingdom

**Keywords:** monkeypox, public health threat, endemic, pandemic, public health action

## Abstract

As the world continues to endure the impact of the COVID-19 pandemic, an outbreak of Monkeypox occurs and continues to spread unabatedly. The double-stranded DNA monkeypox virus is a sylvatic zoonosis, which occasionally infects humans and is a member of the genus Orthopoxviruses. Although scientists believed the virus to have low transmissibility, the speed and degree with which it spreads is alarming and could land one in a hospital or even kill one. Additionally, the fact that unusual transmissions are occurring among people without travel history to endemic regions suggests undetected transmissions, raising concerns about our preparedness for another pandemic. Contrary to the COVID-19 pandemic, there is a vaccine that could offer some protection against the monkeypox virus. Therefore, there is a need for coordinated efforts among authorities concerned and community-based organizations to raise awareness of the potential pandemic of monkeypox, activate surveillance systems and laboratory capacity, and heighten contact tracing and vaccination of at-risk individuals to stem the outbreak while there is still the opportunity to prevent it from becoming a pandemic.

## Introduction

Since early 2020, the world has been facing the fate of the COVID-19 pandemic. While the health systems are still reverting COVID-19, another outbreak of the Monkeypox virus (MPXV) has emerged, spreading unusually. Monkeypox was first discovered in 1958 and the first human case was reported in 1970 in the Democratic Republic of Congo (1). Since then, there have been occasional disease outbreaks, highlighting our healthcare systems' shortcomings.

MPXV is a sylvatic zoonosis, double-stranded DNA virus with occasional human infections that belongs to the family Poxviridae and is a member of the genus Orthopoxviruses (2, 3). It is thought to be transmitted mainly through virus-laced droplets, direct contact with lesions or bodily fluids from an infected person, or indirect contact via contaminated clothing or linens. MPXV usually takes 6–13 days to develop, though it can also take up to 21 days. The condition is frequently self-limiting, with symptoms typically disappearing spontaneously within 14–21 days. Symptoms might be moderate or severe and mostly begins with fever, muscle aches, fatigue, headache, and lesions, which can be itchy or painful. The animal reservoir is unclear; however, it is most likely found among African rats (3). Despite the low case fatality rate of the current outbreak-−20 fatalities have been reported globally—and there have been cases of stigmatization as patients recover and attempt to reintegrate into society (4).

**Figure 1:**
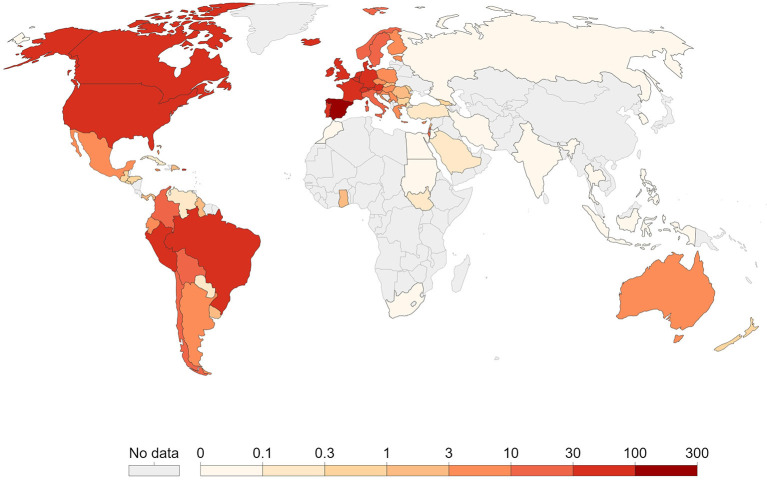
Geographic distribution of monkeypox [our world in data (7)].

**Figure 2:**
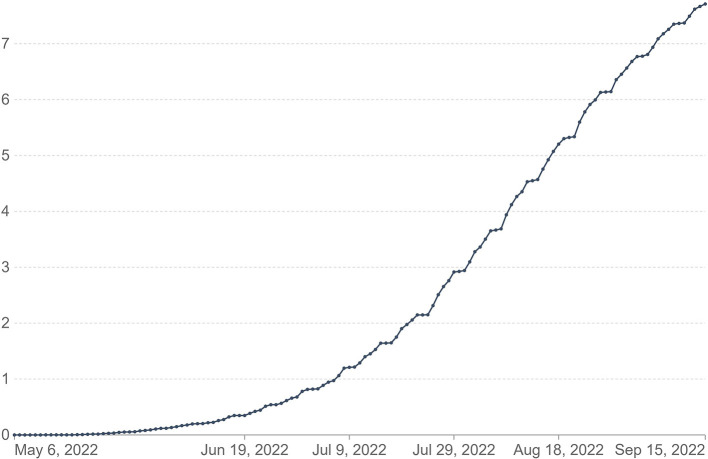
Cumulative confirmed cases of monkeypox per million [our world in data (7)].

As the world is reverting from the impact of COVID-19, several countries reported MPXV outbreaks, which exacerbated issues with already overburdened healthcare systems. The focus on COVID-19 has resulted in significant setbacks in the fight against MPXV. While various national governments raised awareness to strengthen MPXV outbreak prevention and control measures, the necessary resources are already being directed toward the COVID-19 pandemic. As a result, the existing limited resources are put under tremendous strain (5). Whereas, the disease is endemic in Central and Western Africa, the unusually high frequency of transmission observed with this outbreak, as well as the multi-country occurrences without a history of travel to endemic areas, suggests undetected transmissions, which may be due to the long incubation period of MPX compared to COVID-19 (6). This is evidenced by the quick spread of the virus. For instance, as of 15 September 2022, the CDC reported a total of 60,799 cases of MPXV, of which 60,220 were reported in countries with no history of reported cases of MPXV, as shown in Figure 1. Since the outbreak began in the United Kingdom (UK) on 7 May 2022 (7) (see Figure 2), the world has been concerned that if measures are not taken it could lead to another pandemic.

While the smallpox vaccine is believed to be effective against MPX due to earlier studies, it will take a certain period to ramp up production for the entire world. For instance, the US government has ordered 1.6 million doses of the smallpox vaccine, but these will be available at the end of 2022, which would be too late by then given the current spread of the virus (8). Although the smallpox vaccine is up to 85% effective against Monkeypox, most adults in developed countries don't have access to the vaccine, and while someone can get it if infected, it needs to be within 4 days optimally (1). Again, this points back to the COVID-19 pandemic when the wealthy nations stockpile the vaccine while Low and Middle-Income Countries (LMICs) were left behind in protecting their population. Therefore, countries must leverage the lessons learnt from COVID-19 pandemic to scale up testing of MPXV among at-risks population and by extension the general public.

A recent study also raises the possibility that the virus is evolving 12 times more quickly than anticipated. By the end of 2022, the U.K. could see 60,000 new cases per day due to this virus (9). In another study by Verreault et al. (10), they suggest that when in an aerosol, MPX is not easily degraded and can last up to 90 h in suspension. Even though there is still much to learn about the virus, this is not the time to debate on whether we should vaccinate or not, we must take a proactive approach to testing, immunization, and raising public awareness. The WHO should sound the alarm as soon as possible, just as the delay in designating COVID-19 was crucial to the ineffectiveness of controlling its global spread. Otherwise, countless lives will be at risk and we might have to ask, if this becomes a pandemic, who will pay for the vaccine for LMICs?

## What is the public health risk of MPXV?

Scientists believed the virus to have low transmissibility, however; the speed and degree with which it spreads is alarming, and more importantly, it could result in hospitalization or even death. Whereas, many cases of MPXV were identified among Men who have Sex with Men (MSM), there is also reported cases among non-sexual contacts, children, and pregnant women (11). Evidence from the cases detected in this outbreak so far indicates that the likelihood of MPXV spreading to broader populations is extremely low, However, MPX may pose a higher risk among close contacts of MPX cases in very young children, pregnant women, elderly or immune-compromised individuals because of its higher impact on these populations (9). Currently, JYNNEOS (also known as Imvamune or Imvanex) is a live attenuated virus vaccine that has been approved by both the U.S. Food and Drug Administration for the prevention of Monkeypox and by EMA for the EU market against smallpox, but it lacks efficacy data against MPX, and there are no safety studies for smallpox vaccination in children, pregnant women, or the immunocompromised (10), Additionally, there are gaps in the safety profile and efficacy evidence of the existing antiviral medicines for the treatment of potentially serious cases. To determine the severity (morbidity and mortality) of the disease in Europe, more data are still required on the clinical presentation of patients and their outcomes.

## Artificial intelligence (AI) and machine learning (ML) in MPXV surveillance and diagnosis

As artificial intelligence (AI) and Machine Learning (ML) develops rapidly, researchers in the medical field will have new opportunities to explore. Numerous studies have demonstrated that AI and ML can provide promising information about disease trends and can give useful insight into disease control and prevention (12). For example, Yu et al. developed an internet based ML assessment for diagnosis and prevention of chronic diseases (13). Another AI model created by Yu et al. analyses the trend of COVID-19 pandemic transmission (12). Furthermore, different studies have examined different timeframes for predicting pandemic trends. As AI and ML have been demonstrated to be effective in predicting disease trends and diagnosing diseases, a similar application can be adapted to diagnose MPXV-related skin infections that can be diagnosed using images acquired from infected skin. Also, this technology can be used to provide real-time data analyses that can aid in the prevention and control of epidemics.

## Conclusion

The outbreak of MPX presents global health threats. Therefore, the moment has come for the international public health community to acknowledge the urgent need to take action against the MPX virus or we run the risk of another pandemic that the world is unprepared for. There is a need for coordinated efforts among authorities concerned and community-based organizations to raise awareness of the potential pandemic of MPXV, activate surveillance systems and laboratory capacity and heighten contact tracing to stem the outbreak while there is still the opportunity to prevent it from becoming a pandemic. Furthermore, early detection, contact tracing and timely data exchange among health systems are essential components of disease control as the world is not ready for another pandemic.

## Recommendations

The COVID-19 pandemic has shown us how unprepared most health systems worldwide are, ranging from a lack of funding to testing and sequencing of circulating variants, inequitable distribution of life-saving vaccines, a lack of trained personnel, and poor communication among health systems. As the pandemic's impact lingers in the background, all hands must be on deck to ensure the virus is eliminated as the longer it stays with us, the more consequences it may have, such as mutation and greater transmissibility, among other things. As a result, we must employ the full spectrum of prevention and diagnostic measures in order to curb the spread, and to stop the development of local reservoirs in rodents, and prevent suffering and possible deaths. This is especially important for the immune-compromised, pregnant women, and young children. Based on available evidences and international best practices, the following recommendations should be adopted to mitigate the spread of MPX virus:

## Outbreak management

Global efforts should focus on containment and prevention of the further spread of the MPX virus. This can be done through coordinated awareness campaigns on risk management and good hygiene practice. It's imperative that individuals who come in contact with persons or animals suspected of having the MPX virus should practice good hygiene through hand washing with an alcohol-based sanitizer. To stop the virus from spreading continuously, public health authorities and governments around the world must provide public education and support regarding protection measures, rapid case identification, early diagnostics, and contact tracing. This information should be made readily available via social media and billboards in public spaces. Also, the MPX disease is endemic in Western Africa. Therefore, travelers to the endemic region should avoid contact with any animal that may be carriers of the disease (particularly those that are sick or have been found dead).

## Contact tracing and surveillance

Contact tracing has been a cornerstone of public health efforts to combat communicable diseases for many years. For instance, the elimination of smallpox was achieved not just with widespread ring vaccination but also with meticulous contact tracing to identify every contact of sick persons. Therefore, to ensure the public's health continues protection, the national governments of various countries should initiate a massive backward and forward contact tracing program in order to identify individuals who may have come into contact with infected persons. This will ensure that any MPX cases are detected quickly and treated appropriately. Also, diagnostic testing and whole genome sequencing should be done to guide our understanding on the circulating variants and how best to tackle the chain of transmission. Further, it is important to leverage technology such as telemedicine to enhance contact tracing and enable self-reporting of infected individuals.

## Vaccination campaign

Although there is currently no vaccine for monkeypox, smallpox vaccination may provide some protection against MPX due to their genetic similarity. It is therefore essential to vaccinate those who are at risk and those who come into contact with infected people. This is because vaccines are most effective if administered before an infection develops. Healthcare workers represent the cornerstone of the health system, and therefore must be prioritized. Patients with specific medical conditions, such as those who are immune-compromised, young people, or women who are pregnant, shouldn't receive this vaccination. While scientists debated whether the current MPXV is serious enough to warrant mass vaccination, the risk posed by monkeypox to the general public cannot be overstated, and if it becomes a pandemic, the world may face vaccine shortages.

## Data availability statement

The original contributions presented in the study are included in the article/supplementary material, further inquiries can be directed to the corresponding author.

## Author contributions

UH and DL-P conceived the idea. UH, SM, and EM wrote the first draft. AS-S and DL-P reviewed the manuscript with significant scholarly contribution. All authors read and approved the manuscript.

## Funding

This project is funded by Nazarbayev University under Project Reference: 021220CRP0822.

## Conflict of interest

The authors declare that the research was conducted in the absence of any commercial or financial relationships that could be construed as a potential conflict of interest.

## Publisher's note

All claims expressed in this article are solely those of the authors and do not necessarily represent those of their affiliated organizations, or those of the publisher, the editors and the reviewers. Any product that may be evaluated in this article, or claim that may be made by its manufacturer, is not guaranteed or endorsed by the publisher.
